# Viromes As Genetic Reservoir for the Microbial Communities in Aquatic Environments: A Focus on Antimicrobial-Resistance Genes

**DOI:** 10.3389/fmicb.2017.01095

**Published:** 2017-06-15

**Authors:** Stefano Colombo, Stefania Arioli, Eros Neri, Giulia Della Scala, Giorgio Gargari, Diego Mora

**Affiliations:** Department of Food, Environmental and Nutritional Sciences, University of MilanMilan, Italy

**Keywords:** virome, microbiome, antimicrobial resistance genes, mesocosms, Lambro River

## Abstract

Despite studies of viromes isolated from aquatic environments are becoming increasingly frequent, most of them are limited to the characterization of viral taxonomy. Bacterial reads in viromes are abundant but the extent to which this genetic material is playing a role in the ecology of aquatic microbiology remains unclear. To this aim, we developed of a useful approach for the characterization of viral and microbial communities of aquatic environments with a particular focus on the identification of microbial genes harbored in the viromes. Virus-like particles were isolated from water samples collected across the Lambro River, from the spring to the high urbanized Milan area. The derived viromes were analyzed by shotgun metagenomic sequencing looking for the presence, relative abundance of bacterial genes with particular focus on those genes involved in antimicrobial resistance mechanisms. Antibiotic and heavy metal resistance genes have been identified in all virome samples together with a high abundance of reads assigned to cellular processes and signaling. Virome data compared to those identified in the microbiome isolated from the same sample revealed differences in terms of functional categories and their relative abundance. To verify the role of aquatic viral population in bacterial gene transfer, water-based mesocosms were perturbed or not perturbed with a low dose of tetracycline. The results obtained by qPCR assays revealed variation in abundance of *tet* genes in the virome and microbiome highlighting a relevant role of viral populations in microbial gene mobilization.

## Introduction

Studies of the environments through metagenomics analysis are becoming increasingly frequent and the development of instruments, techniques and databases makes the study and the characterization of viral communities with a shotgun metagenomics approach more and more informative. Water has been described as the major environment for bacteria on earth (Taylor et al., 2011), and bacteriophages outnumber bacteria by a factor ranging from 1 to 10 (Muniesa et al., 2013a). Water is an ideal medium for bacterial life, characterized by accessible dissolved nutrients, as well as protection from desiccation and UV light. In this context, the high frequency of encounters between bacteria and bacteriophages strongly favors, through transduction, the mobilization of genetic material. Most studies focused on environmental viromes are limited to the characterization of viral taxonomy (Roux et al., 2012; Weynberg et al., 2014; Danovaro et al., 2016). However, viromes harbor huge amounts of bacterial reads which account for up to 90% of the microbial reads (Fancello et al., 2013; Modi et al., 2013; Zablocki et al., 2014; Abeles et al., 2015). Few studies reported that phage metagenomes were enriched with microbial functional genes (Fancello et al., 2013; Modi et al., 2013; Colombo et al., 2016), which clearly reported that phage metagenomes were enriched with microbial functions. The extent to which these virome-associated microbial genes are accessible to members of the environmental microbiota remains unclear. In this context, those factors triggering the mobilization and spreading of microbial genes by the viral communities in water environments are still processes not completely understood. To date the attention was mainly focused on the mobilization of antibiotic-resistance genes (ARGs) (Allen et al., 2010; Muniesa et al., 2013a,b). A part from the water environments, the role of viruses in mobilization of ARGs in the human and mice microbiota after exposure to antibiotics has been recently experimentally documented, showing that the treatment with antibiotics leads to an increase in the ARGs abundance in the virome (Fancello et al., 2013; Modi et al., 2013; Abeles et al., 2015).

Rivers can be polluted by a diverse mixture of antibiotics and other pollutants, metabolites and resistant bacteria through treated and untreated sewage, hospital waste, aquaculture discharges, and agricultural run-off. These aquatic compartments, such as water and sediment, may therefore have a significant role in driving ARGs transfer, ecology and evolution (Taylor et al., 2011; Marti et al., 2014). In this work, we developed a method for the comprehensive characterization of viral and microbial communities by shotgun metagenomics focusing the attention on the presence and relative abundance of microbial gene sequences in viromes isolated from the Lambro River flow, from the spring to the high urbanized/industrialized Milan area. The aim of this work was to describe the relative abundance and the classification in functional categories of the bacterial gene sequences associated with aquatic environment viromes. A special attention was payed to the genes associated with the antimicrobial-resistance mechanisms such as ARGs and heavy metal resistance genes. With the aim to prove the role of viruses in the mobilization of ARGs, tetracycline-resistance (*tet*) genes were monitored in Lambro River water-based mesocosms, perturbed and non-perturbed with a low dose of the antibiotic tetracycline.

## Materials and Methods

### Sampling Procedures

Lambro River catchment is located in the north of Milan with a total drainage area of approximately 1,950 km^2^. Lambro River’s spring is situated in the Pre-Alps (1,450 m above sea level) in the Magreglio area, and it flows into a confluence with the Po River (50 m above sea level) with an estimated length of 130 km. The River catchment average annual rainfall varies between 900 and 1,500 mm (Deksissa et al., 2004).

Three water samples (30 L) were collected in February 2015 along Lambro River. The first sample was collected at the river’s spring in Magreglio area (45.935270, 9.264576). This sample due to the characteristics of the area can be considered as belonging to a no urban impact area (A). The second sampling point was near the town of Ponte Lambro (45.49349, 9.13274) approximately 15 km from the Lambro River origin (B). The third sampling point was in Milan in Lambro Park (45.495399, 9.247936) approximately 55 km from the origin (C). Samples were maintained at 6°C for 4 h before processing.

### Virus-Like Particle Isolation

Water samples were filtered at 0.2 μm (Pall, Life Sciences, Milan, Italy) with a tangential flow filtration system (TFF) (Quattro systems, Parma, Italy), as previously described in Colombo et al. (2016). The permeate collected after the 0.2 μm TFF, containing all the virus-like particles (VLPs), was subsequently ultrafiltered at 100 kDa (Pall, Life Sciences, Milan, Italy) using the same TFF system; pressure was maintained under 62 kPa in order to avoid VLP damage (Thurber et al., 2009). The retained material containing the VLPs (400 mL) was then precipitated overnight at 4°C using PEG 8000 at a final concentration of 10% (wt/vol) and subsequently centrifuged at 13,000 × *g* for 30 min at 4°C. The pellet was suspended in TE buffer (pH 8.0) and prepared for CsCl gradient centrifugation. The solution was then deposited on top of a 2.5 mL step gradient composed of multiple 0.8 mL CsCl solutions with corresponding densities of 1.70, 1.50, and 1.35 g mL^-1^. Samples were centrifuged for 2 h at 60,000 × *g* (4°C) in a SW41 swinging bucket rotor (Beckman Instruments Inc., Fullerton, CA, United States) (Thurber et al., 2009). According to the protocol proposed by Thurber et al. (2009), we recovered approximately 2 mL of the 1.5 g mL^-1^ layer, because material in this density range should be enriched with VLPs.

### Extraction and Amplification of VLP DNA for Metagenomic Analysis

Viral DNA was extracted according to a protocol previously described by Thurber et al. (2009). The 1.5 g mL^-1^ layer collected from the step gradient was treated with DNase (37°C, 12 h), following the manufacturer’s instructions (Sigma–Aldrich, Milan, Italy; final concentration, 2.5 U mL^-1^), to remove residual host and bacterial DNA. To extract the virions, 0.1 volumes of 2 M Tris HCl/0.2 M EDTA, 1 volume of formamide and 100 μL of a 0.5 M EDTA solution was added to the 10 mL sample, and the resulting mixture was incubated at 25°C for 30 min. The sample was subsequently washed with 2 volumes of 70% ethanol and pelleted by centrifugation at 8,000 × *g* for 20 min at 4°C. The pellet was washed twice with 70% ethanol and resuspended in 567 μL of TE buffer, followed by the addition of 30 μL of 10% SDS and 3 μL of a 20 mg mL^-1^ solution of proteinase K (Fisher Scientific, Waltham, MA, United States). The mixture was incubated for 1 h at 55°C and supplemented with 100 L of 5 M NaCl and 80 μL of a solution of 10% cetyltrimethylammonium bromide/0.7 M NaCl. After a 10 min incubation at 65°C, an equal volume of chloroform was added, and the mixture was centrifuged (5 min at 8,000 × *g* at 25°C). The resulting supernatant was transferred to a new tube, and an equal volume of phenol/chloroform/isoamyl alcohol (25:24:1) was added. The mixture was centrifuged (5 min at 8,000 × *g* at 25°C). The supernatant was recovered, and an equal volume of chloroform was introduced. Following centrifugation, the supernatant was collected, and 0.7 volumes of isopropanol were used to precipitate the DNA. After centrifugation (15 min at 13,000 × *g* and 4°C), the DNA pellet was washed with 500 μL of ice-cold 70% ethanol, air-dried and resuspended in 50 μL TE buffer (Thurber et al., 2009).

In order to increase the amount of DNA available, whole-genome amplification was performed using reagents and protocols of the Illustra GenomiPhi V2 kit (GE Healthcare, Milan, Italy) to generate sufficient material for metagenomic analysis. Purified VLP DNA (5–15 ng) was mixed with 9 μL of sample buffer provided by Illustra GenomiPhi V2 kit and heat-denatured at 95°C for 3 min. Subsequently, 9 μL of kit reaction buffer and 1 μL of kit enzyme mix were added, and the solution was incubated for 90 min at 30°C. The amplified products were subsequently pooled and purified (UltraClean PCR Clean-UP Kit, MoBio Laboratories, Solana Beach, CA, United States) (Thurber et al., 2009). Finally, 2 μL of each DNA solution was quantified using the PowerWave XS Microplate Spectrophotometer at 260 nm (BioTek Instruments, Inc., Winooski, VT, United States) and the Take3 Multi-Volume Plate (BioTek Instruments, Inc., Winooski, VT, United States).

### Calculation of Bacterial Contamination Using qPCR

An aliquot of the purified DNA was used as a template in qPCR assays to quantify the amount of contaminating, non-viral DNA. A CFX96 thermocycler (Bio-Rad Laboratories S.r.l., Milan, Italy) was used to quantify the bacterial DNA. The analysis was performed using a Fast Eva Green Supermix SYBR Green PCR Master Mix (Bio-Rad Laboratories) in a reaction volume of 15 μL per well. qPCR amplification was carried out with an initial denaturation at 95°C for 5 min, followed by 40 cycles of denaturation at 95°C for 30 s, annealing at 58°C for 30 s and extension at 72°C for 30 s. The primers EubF1 (5′-GTGSTGCAYGGYTGTCGTCA-3′) and EubR1 (5′-GAGGAAGGTGKGGAYGACGT-3′) were used to target *16S rRNA* to detect any eubacteria. Data were recorded as threshold cycles (*C_tT_*) and expressed as mean values; standard deviations were computed using the Bio-Rad CFX Manager. DNA from pure cultures of *Lactococcus garvieae* strain TB25 was subjected to six 10-fold serial dilutions and used as templates for qPCR. Standard curves were generated by plotting the log_10_ of the bacterial cell numbers against the corresponding C*_t_* values obtained from the amplification of diluted DNA. Calibration curves showed good correlation between C*_t_* values and the number of cells over the considered range (*r*^2^ regression coefficients were between 0.964 and 0.999). The resulting detection limits were between 1.1 and 1.9 log_10_ bacterial cells per reaction mix (Guglielmetti et al., 2013). On the basis of the C*_t_* values obtained using viral DNA, the actual concentration of bacterial DNA was determined and expressed as a percentage of the total DNA previously extracted. Bacterial contaminating DNA accounted for less than 0.2% of the total viral DNA extracted.

### Extraction of Microbial DNA for Metagenomic Analysis

Water samples were filtered at 0.2 μm by TFF, and the obtained permeate was centrifuged for 15 min at 9000 × *g* and 4°C. The biomass obtained was then suspended in 400 μL of TE buffer (pH 8). To this purpose, 15 μL of lysozyme (20 mg mL^-1^) was added before a 60 min incubation at 37°C. Subsequently, 15 μL of SDS (20%) and 15 μL of proteinase K (20 mg mL^-1^) (Fisher Scientific) were added, and the mixture was incubated at 55°C for 15 min. The mixture was supplemented with 250 μL of phenol and centrifuged at 22,000 × *g* for 5 min. The phenol phase was removed, and 400 μL of chloroform was added to the aqueous supernatant. The centrifugation step was repeated as described above. The supernatant was transferred to a new tube with 40 μL of sodium acetate (3 M, pH 5.2) and 800 μL of 96% ethanol (-20°C). The mixture was centrifuged for 30 min at 22,000 × *g* and 4°C. The liquid was discarded, and the pellet was washed with 300 μL of 70% ethanol (-20°C). The centrifugation step was repeated as described above, and the liquid was discarded. The DNA was dried and suspended in 40 μL of sterile distilled water. The PowerWave XS Microplate Spectrophotometer at 260 nm (BioTek) and the Take3 Multi-Volume Plate (BioTek) were used to quantify 2 μL of the DNA solution.

### Metagenomic Shotgun Sequencing of Microbial and Viral DNA

A genomic library was generated from 3 μg of genomic DNA using the TruSeq DNA PCR-Free Sample Preparation Kit (Illumina) and the MiSeq Reagent, according to the user’s guide (Illumina). The quality of the library was estimated prior to loading onto a flow cell, and the library sample was subsequently sequenced using 500 sequencing cycles, according to the MiSeq (Illumina) instructions. The 500 sequencing cycles resulted in an average read length of approximately 250 nucleotides for both paired-end sequences. The MIRA program version 4.0.2 (Chevreux et al., 1999) was used for *de novo* assembly of contigs. Mira options were set as follows: “job = genome, *de novo*, accurate parameters = -nw:cac = no -ge:not = 1 iontor_settings -as:mrpc = 100” ([minimum_reads_per_contig (mrpc) = 100]). Assembled reads were searched for ORFs predicted with PRODIGAL v2_60 linux^1^ (Hyatt et al., 2010).

#### Quality Control of Virome Reads

Reads from metagenomic shotgun sequencing were initially checked for quality using the FASTQC program^2^. Fastq files were trimmed and filtered with FASTX-Toolkit^3^ and homerTool^4^. The fastq output was filtered for reads with a quality of < 25, as well as reads of 80 bp. Bases were removed from the ends of the reads until the average quality in a window of 5 bp was > 25. Contaminant reads (i.e., human reads) were removed with CS-SCORE (Haque et al., 2015). Sequencing metadata, assembly metrics, and annotation statistics are summarized in Supplementary Table S1.

Identification of contaminant reads in viral metagenomes was performed with a BLASTn analysis on the “16SMicrobial” database provided by NCBI^5^. BLASTn was performed with a threshold of 10^-5^ on the *E*-value, and only results with the identity over 90% were kept. Levels of contamination were then expressed in percentage (%) as a number of reads identified with the “16SMicrobial” database over the total number of reads.

### Bioinformatics

A BLAST analysis was performed against non-redundant viral database using BLAST+ 2.2.29 ftp://ftp.ncbi.nlm.nih.gov/blast/executables/blast+/LATEST/. A database was created using all entries for the taxonomy ID 10239 (Viruses) on NCBI.

Both viral and microbial data underwent a BLAST analysis against both the non-redundant nucleotide and the non-redundant protein database of NCBI; BLASTn, BLASTp, and BLASTx were performed with a threshold of 10^-5^ on the *E*-value. In order to identify reads associated to ARGs, the data were compared with the CARD (Comprehensive Antibiotic Resistance Database); the database is composed of 3228 genes specifically tagged for antibiotic resistance (McArthur et al., 2013). BLASTp and BLASTn were performed in order to identify the antibiotic resistance-encoding reads with a threshold of 10^-5^ on the *E*-value. Only sequences with an amino acid identity > 30% and a nucleotide identity > 70% were considered.

Identification of metal resistance reads was performed with BLASTp and BLASTn against BacMet database (Pal et al., 2014). All bioinformatics analyses were performed using the CINECA SCAI (SuperComputing Applications and Innovation) (CINECA, Bologna, Italy), part of the ISCRA VArFaWtr project.

#### Accession Numbers

The sequence data were submitted to the EMBL-EBI database under the accession number PRJEB14951.

### Evaluation of Antibiotic Resistant Colony Frequencies

For the evaluation of antibiotic resistant colony frequencies, samples from the 0.2 μm TFF system retentate (see next paragraph) were used for dilution and plating on two selective media: plate count agar (PCA) and MacConkey (MC) agar (Sigma–Aldrich). Both media were also tested with the addition of antibiotics such as ampicillin, tetracycline, chloramphenicol, and meropenem. The antibiotics were present at a final concentration of 20 μg mL^-1^. Samples were incubated at 20°C for 48 h. The microbial count was carried out in triplicate.

### Monitoring of *tet* Genes in Perturbed and Non-perturbed Mesocosms

To understand the role of viral population in the mobilization of *tet*, we prepared mesocosms using water collected from the Lambro River at the Milan sampling site. In detail, the Lambro River water-based mesocosms were prepared with and without the addition of tetracycline used as a selective pressure agent. Because tetracycline resistance-related reads were identified in all three viromes analyzed, and due to the availability of qPCR protocols for detection and quantification of *tet* genes, we choose this antibiotic as a bacterial perturbation agent in the mesocosms. All mesocosms were prepared in 20 L sterilized tanks maintained under static conditions, at environmental temperature (6–10°C), and not exposed directly to the sun light. After sampling the water from the river, eight mesocosms were maintained in the above-described condition for 48 h. After that period, two mesocosms were supplemented with tetracycline (Sigma–Aldrich) at a final concentration of 100 ng L^-1^ and other two mesocosms were used as controls. After 24 h all four mesocosms were treated to collect the viral populations by TFF as previously described. The viral populations collected, were subjected to further purification of VLP and DNA purification as described above. The 0.2 μm TFF retentate, containing the microbiota, was used for the extraction of total DNA as described above. Viral and microbial DNA were used as templates for the qPCR assay to quantify genes associated with tetracycline resistance (*tet* genes) according to the protocols described by Yu et al. (2005) and Fan et al. (2007). After perturbation with 100 ng L-1 of tetracycline, the virome and microbiome were collected, and a set of *tet* genes coding for Gram-negative efflux pumps (tetA-C, tetE, tetG, and tetH) (Pao et al., 1998; Yu et al., 2005; Fan et al., 2007) were quantified by qPCR. qPCR assays were performed in a CFX96 thermocycler (Bio-Rad Laboratories). The reaction mixture was prepared using a Fast Eva Green Supermix SYBR Green PCR Master Mix (Bio-Rad Laboratories) in a reaction volume of 15 μL per well, using 5 ng of viral or microbial DNA as the template. All qPCR assays were performed in triplicate. The quantification of *tet* genes was shown as the average of the *C_t_* values ± the standard deviation. Statistically significant differences were determined by an unpaired Student’s *t*-test (^∗^*P* < 0.05).

## Results

### Taxonomic Description of Viral Communities Using the Shotgun Metagenomic Approach

**FIGURE 1:**
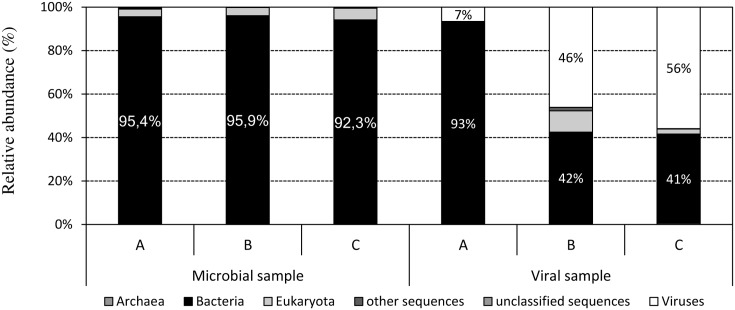
Taxonomic affiliations at phylum levels of the reads from metagenomic shotgun sequencing in microbial and viral metagenomes, assigned by BLAST with a threshold of 10^-5^ for the *E*-value against the NCBI taxonomy database.

Identification of viral reads in the viromes of the three samples resulted in two different scenarios (**Figure 1**). The sample A was characterized by the largest abundance of bacterial reads (93%), and only 7% were identified as viral reads. On the contrary, the other two samples showed a lower amount of bacterial reads (41–42%) and a higher level of the viral reads (46–56%). In addition, we would like to underline that ribosomal sequences were detected with a frequency (no. of ribosomal reads on the total of reads in virome) largely below the 0.2‰ threshold in two of the three viral metadata. Currently, when > 0.2‰ of reads match to 16S ribosomal DNA, a non-viral marker of bacterial DNA, the viromes are considered to have high bacterial DNA content (Roux et al., 2013). We obtained the level of 0.006 and 0.009‰ of reads match to 16S ribosomal DNA for samples B and C, and 0.35‰ for sample A. Moreover, in all three samples qPCR quality control assays revealed that the levels of bacterial *contaminating* DNA in virome samples accounted for less than 0.2% of the total viral DNA extracted, a threshold considered acceptable as suggested by Modi et al. (2013). We therefore exclude that this low level of bacterial DNA contamination in viromes could have affected the subsequent bioinformatic analysis of viromes.

**FIGURE 2:**
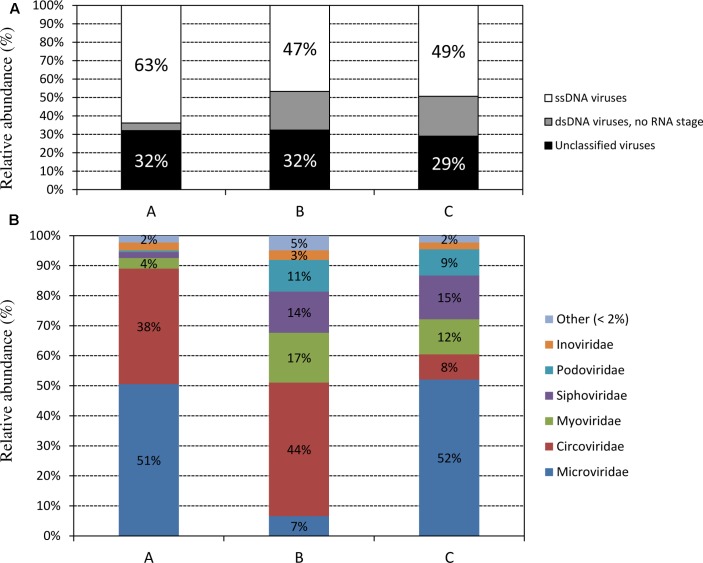
Taxonomic affiliations at kingdom **(A)** and family **(B)** levels of the reads from metagenomic shotgun sequencing of the three viral samples, assigned by BLAST against our customized viral NCBI taxonomy database.

In our samples taxonomy was first associated with the viral reads. Viral reads were mainly identified as ssDNA viruses and as dsDNA viruses (**Figure 2A**), as expected for bacteriophages. Among them approximately 30% of the reads were classified only at the kingdom level. The remaining reads showed a taxonomy distribution as follows: *Microviridae* was the most abundant family identified both in samples B (51%) and C (52%) while only 7% in sample A. Similarly, *Circoviridae* were present in a large amount in samples A and B (38 and 44%, respectively), while only the 8% of the identified viral reads were recognized in sample C. To a lesser extent *Myoviridae* and *Siphoviridae* were present in a similar abundance in samples A and C (17 and 12%; 14 and 15%, respectively) while only at a low amount in the sample B (4 and 2%) (**Figure 2B**).

**FIGURE 3:**
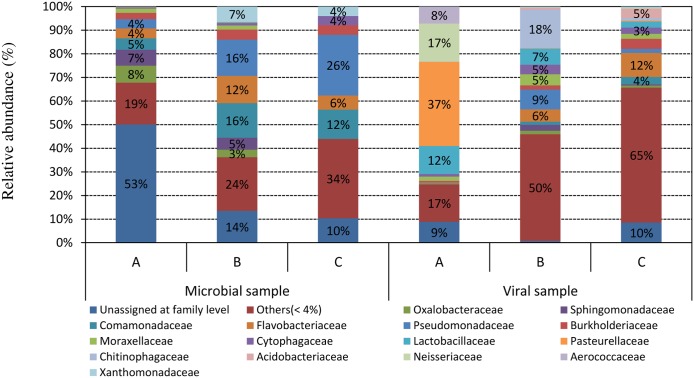
Taxonomic affiliations at family levels of the reads from metagenomic shotgun sequencing in microbial and viral metagenomes, assigned by BLAST with a threshold of 10^-5^ for the *E*-value against the NCBI taxonomy database.

A separate taxonomic identification of the microbial reads in the three viromes has been performed (**Figure 3**). As observed by other authors (Rosario et al., 2009; López-Bueno et al., 2009; Roux et al., 2012; Fancello et al., 2013; Modi et al., 2013) bacterial reads in viromes are abundant and accounted for up to 90% of the microbial reads. We therefore focused the attention on their taxonomic identification. *Flavobacteriaceae* was the only family that stood out of the three samples (37% in the A sample in comparison to 6 and 12% in samples B and C, respectively). Other notable families identified were *Chitinophagaceae* (18% in sample B), *Neisseriaceae* (17% in sample A), and *Lactobacillaceae* (12% in sample A). Virome of sample C was the richest regarding bacteria with 294 different bacterial families represented, followed by viromes of samples A and B showing 290 and 236 bacterial families, respectively.

### Functional Characterization of Virome Data

**FIGURE 4:**
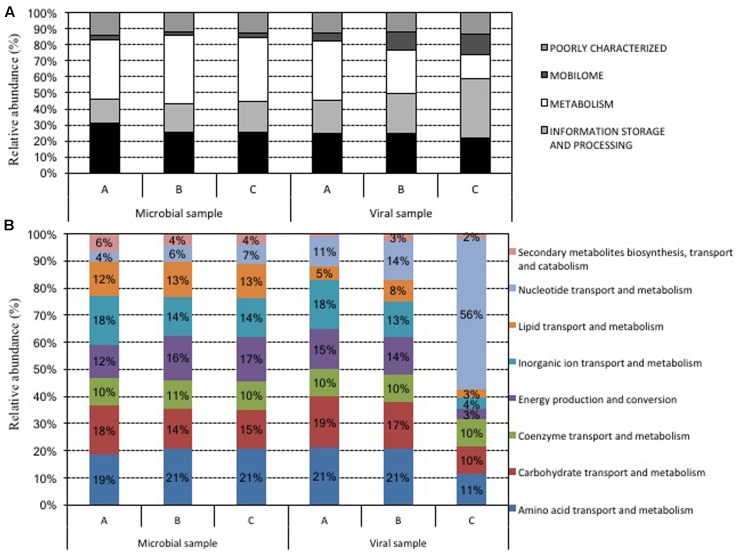
Functional classification of the reads from metagenomic shotgun sequencing of the microbial and viral samples. The data were first assigned to general classes with the COG database **(A)**, then attention was focused on metabolism-related classes **(B)**. Data were expressed as relative abundance with respect to the total reads **(A)** and with respect to only reads belonging to metabolism-related classes **(B)**.

Metabolism-related reads in viromes showed a range of abundance between 15 and 37%, whereas information storage and processing-related reads ranged between 20 and 36% (**Figure 4**). The abundance of reads assigned to cellular processes and signaling was similar in all three samples (25-25-22%). Focusing the attention at the subcategories (**Figure 4B**), we observed a similar distribution of WHAT? in all samples for the most of them with the exception of nucleotide transport and metabolism-related reads, which accounted for up to 56% in sample C (11 and 14% in the other two samples).

Mobilome-associated reads showed an abundance ranged between 5 and 12%. This category, as defined by NCBI-COG, mainly groups reads that encode for phage-related proteins as terminase, capsid, tail and other as transposase and protein involved in initiation of plasmid replication. In detail, phage-related ORFs accounted for 20–59% of the mobilome, whereas transposase and protein involved in plasmid replication showed a relative abundance between 33 and 37% of the mobilome.

### Functional Characterization of Microbiome Data

As for analysis of virome, functional classification of microbiome reads was performed using the COG database. No changes were observed for each functional category among the different samples (**Figure 4A**). Metabolism-related reads were the most represented (37–42%), reads associated with “cellular processes and signaling” were the second most observed (25–31%), and reads related to “information storage and processes” were the third most observed (15–19%) (**Figure 4A**). Reads included in the “mobilome” category accounted for 2–3% of the total: among these, up to 65% were identified as transposase-related reads, while from 12 to 25% were classified as phage-related reads. Among the metabolism-related category, none was predominant with respect to the others: amino acid transport and metabolism (19–21%), carbohydrate transport and metabolism (14–18%), inorganic ion transport and metabolism (14–18%), lipid transport and metabolism (12–13%), and energy production (12–17%) (**Figure 4B**).

### Identification of Antibiotic Resistance Genes in the Microbiome and Virome

**FIGURE 5:**
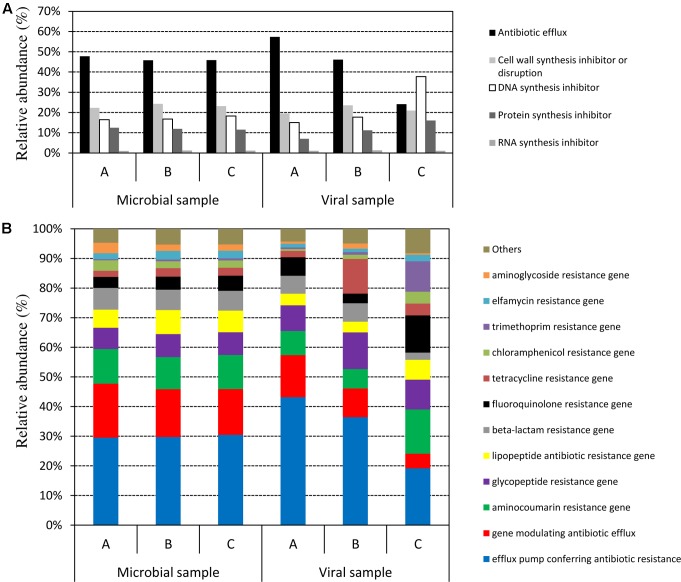
Comparison of the distribution of antibiotic resistance-related reads among the microbial and the viral fraction of the three samples. **(A)** Distribution of the drug classes based on the inhibition target of the antibiotic. **(B)** Distribution of reads identified as antibiotic-resistance genes (ARGs) expressed as relative abundance with respect to the total ARGs-related reads identified.

In viromes we observed a relative abundance of reads associated with ARGs ranging from 0.48% (sample C) to 1.92% (sample A) (calculated as the relative abundance of reads associated with ARGs over the total reads identified within NCBI) (**Figure 5A**). The genes coding for DNA synthesis inhibitors and protein synthesis inhibitor classes showed an abundance ranging from 15 to 38%, whereas ARGs belonging to efflux pumps and genes modulating antibiotic efflux ranged between 24 and 57%. Aminocoumarin and glycopeptide resistance genes were two of the most represented in the three samples. Tetracycline-resistance genes ranged between 2 and 12% (**Figure 5B**).

In microbiome, the relative abundance of reads associated with ARGs ranged between 8 and 12% (calculated as the relative abundance of reads associated with ARGs over the total reads identified within NCBI). Nevertheless, the three microbiomes showed a similar distribution of the five drug classes as shown in **Figure 5A** (here the relative abundance is calculated for the total amount of reads associated with ARGs): ARGs that encode efflux pumps were the most abundant (∼47%), followed by cell wall synthesis and disruption ARGs (23%), and finally DNA synthesis inhibitors (17%). Looking specifically at the antibiotic resistance genes identified from the microbial metagenome, an identical situation was observed: up to 45% of the ARGs identified were genes that encode for efflux pumps or genes modulating the efflux systems (**Figure 5A**). As previously described for viromes, aminocoumarin, and glycopeptide resistance genes were the most represented in all three microbiome samples with amounts ranging from 12 to 16%. Sequences belonged to beta lactam coding genes, and chloramphenicol and tetracycline-resistance genes were also found (**Figure 5B**).

### Identification of Heavy Metal and Biocide Resistance Genes

From the spring to Milan area, the Lambro River crosses increasingly industrialized areas that negatively affect the point-source pollution levels in its waters, specifically pesticides, metals, organochlorines, and organic compounds. For these reasons, microbiomes and viromes were analyzed for the identification of genes related to resistance mechanisms against heavy metals (MRGs). In the microbiomes, the relative abundance of MRGs ranged from 9% in sample A to 14% in sample C. In viromes, MRGs ranged between 3% in sample A to 1% in sample C. Fifty-six different resistance entries were identified with a similar pattern of relative abundance among the six different metagenomes (Supplementary Table S2). Among the genes associated with metal resistance the highest abundances identified were against copper (∼13% in the three microbiomes while up to 20% in virome sample B), zinc (an average of 13% in the microbiomes and from 8 to 14% in the viromes), iron (8% in the microbiomes; up to 16% in virome sample A), and nickel (7% in the microbiomes; from 5 to 13% in the viromes). Biocide resistance genes were identified in a much lower amount with respect to the MRGs: among them the most abundant identified were resistance genes against hydrogen peroxide (2% in the microbiomes and up to 4% in the viromes).

### Frequency of Antibiotic Resistant Colonies in Water Samples

**FIGURE 6:**
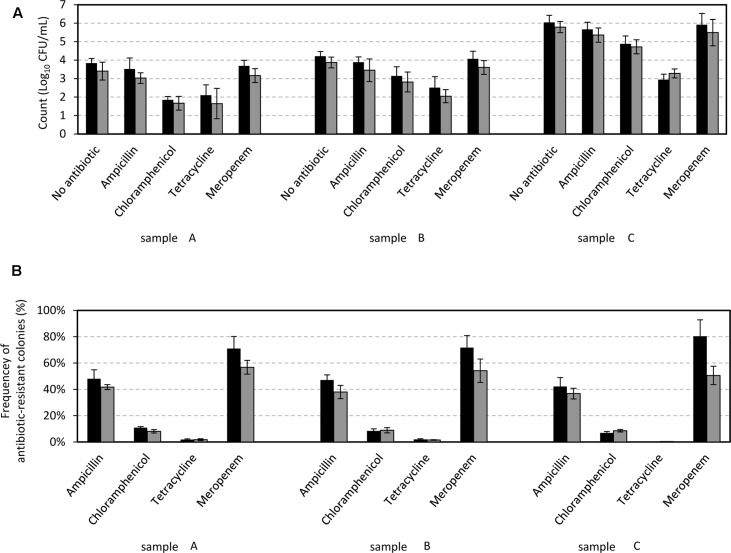
The microbial count in water samples, expressed as Log_10_ (CFU/mL). The microbial count was carried out in plate count agar (PCA) media (black bars) and MacConkey (MC) media (gray bars) after incubation at 20°C for 48 h in the absence of antibiotics **(A)**, and with the addition of antibiotics at a final concentration of 20 μg/mL. Frequency of the antibiotic-resistant colonies **(B)** was calculated as the ratio of the colony growth in the presence of a specific antibiotic compared to colony growth in its absence. Numbers are expressed as the percentage ± the standard deviation.

With the aim to evaluate the frequency of antibiotic resistant colonies of the aquatic microbial communities, we first quantified the total cultivable microbial abundance by plating on two different media: PCA and MC. The total bacterial count obtained revealed an increase of cultivable bacteria from the spring to the water samples collected close to Milan city. On PCA, the bacterial count ranged between 7 × 10^3^ CFU L^-1^ (3.8 log10) ± 4 × 10^2^ in sample A and 1 × 10^6^ Log_10_CFU L^-1^ (6.1 log10) ± 7 × 10^4^ in sample C (**Figure 6A**). Similarly, on MC agar the Gram-negative bacterial count ranged between 2 × 10^3^ CFU L^-1^ (3.4 log10) ± 4 × 10^2^ in sample A and 6 × 10^5^ CFU L^-1^ (5.8 log10) ± 3 × 10^4^ in sample C. Focusing on the frequencies of the antibiotic resistant colonies, surprisingly no significant differences were observed among the different samples. In PCA media the highest frequencies were observed in presence of beta lactamic antibiotics: meropenem-resistant colonies reached up to 70% of the total, and ampicillin-resistant colonies were approximately 47%. However, chloramphenicol-resistant colonies accounted for 7–11%, and tetracycline-resistant colonies were under 2% in all three samples (**Figure 6B**). We therefore conclude that the frequency of antibiotic resistant colonies was not affected by the increasingly industrialized and urbanized areas crossed by the Lambro River from samples A to C. However, the absolute number of antibiotic-resistant colonies increased significantly from samples A to C area as a consequence of the increase in the total bacterial count in the water samples.

### Changes in *tet* Genes in Virome- and Microbiome-Exposed and Non-exposed to Low Dose of Tetracycline in Lambro River Water-Based Mesocosms

**FIGURE 7:**
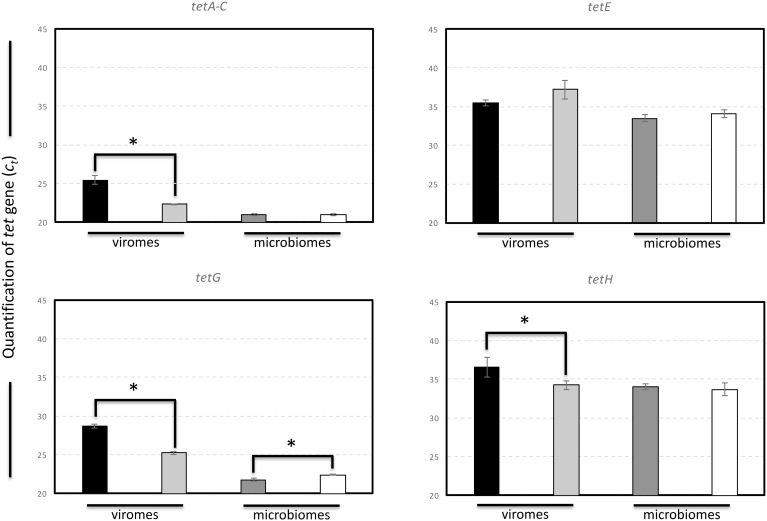
Quantification of *tet* genes in viromes and microbiomes isolated from Lambro River water-based mesocosms. Black bars refer to the *C_t_* values obtained for quantification of *tet* genes in viromes isolated from tetracycline-exposed mesocosms. Gray bars refer to the *C_t_* values obtained for quantification of *tet* genes in viromes isolated from control mesocosms. Dark gray bars refer to the *C_t_* values obtained for quantification of *tet* genes in microbiomes isolated from tetracycline-exposed mesocosms. White bars refer to the *C_t_* values obtained for quantification of *tet* genes in microbiomes isolated from control mesocosms. *C_t_* values are expressed as the average of three independent qPCR assays ± the standard deviation. Statistically significant differences were determined by an unpaired Student’s *t*-test (^∗^*P* < 0.05).

With the aim of following changes in tetracycline-resistant gene (*tet*) in the viromes and microbiomes, we prepared mesocosms using water collected from the Lambro River in the Milan sampling site. The results obtained revealed a significant reduction of *tetA-C*, *tetG*, and *tetH* genes (higher *C_t_* values) in viromes collected from mesocosms that were exposed to tetracycline compared to *tet* genes quantified in viromes collected from non-perturbed mesocosms (lower *C_t_* values), used as control. Interestingly, no significant changes in the abundance of *tet* genes were detected in microbiomes with the exception of *tetG*, which was higher in the microbiomes collected from tetracycline-exposed mesocom compared to the controls. No significant changes in the frequency of tetracycline-resistant colonies were detected between the two mesocosms after tetracycline addition. The overall data obtained suggested that the environmental perturbation with tetracycline determined a mobilization of *tet* genes, the majority of which decreased in viral populations. Conversely, an increase of *tet* genes in the microbiomes was detected only for *tetG* (**Figure 7**).

## Discussion

The role of viruses in controlling and shaping microbial communities is getting more and more evident (Modi et al., 2013; Muniesa et al., 2013a,b; Danovaro et al., 2016). In most of these studies viromes were characterized in terms of abundance of VLPs, and in terms of relative abundance of the taxonomical classes of viruses, without a deep description of the microbial genes harbored by viral genomes in terms both of taxonomy and function. Some authors hypothesize that phageome may represent a community-based mechanism for protecting microbial community, preserving its functional robustness during environmental stress (Modi et al., 2013). Modi et al. (2013) explored the murine gut phageome as a potential genetic reservoir for bacterial adaptation following antibiotic treatment. The authors observed that antibiotic treatment leads to the enrichment of phage-encoded genes that confer resistance via disparate mechanisms to the administered drug. Based on all these considerations, we decided to explore the quality and abundance of microbial gene sequences in aquatic viromes isolated from river samples in order to set up a protocol suitable to describe the phage-bacterial ecological network. Lambro River was chosen as a model because it is known as a high environmental risk area for more than 20 years due to contamination with heavy metals and persistent organic pollutants. In all water samples collected we were able to isolate and concentrate the microbiota and the viral populations adopting a TFF-based procedure. A CsCl gradient centrifugation followed by a DNAse treatment of concentrated VLPs limited under the 0.2% threshold the contamination (qPCR evaluated) of bacterial DNA in viral DNA extraction. This extremely low level of contamination was reflected, after viral genome amplification and shotgun sequencing, in values lower than 0.2‰ of abundance of reads associated to ribosomal genes on the total of reads in two out of three viromes analyzed. These values of contamination are comparable and sometimes lower than those measured in viromes already available in public database (Supplementary Table S3) (López-Bueno et al., 2009; Rosario et al., 2009; Fancello et al., 2013; Zablocki et al., 2014).

**FIGURE 8:**
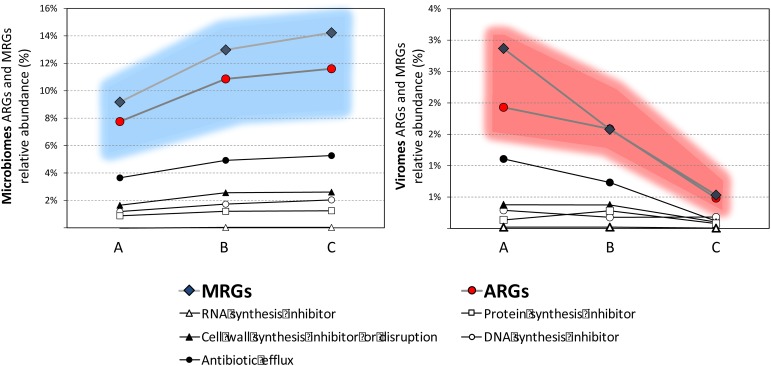
Frequency of ARGs and MRGs in the total amount of reads in the microbiomes (highlighted in the blue-shaded area) and viromes (highlighted in the red-shaded area). The frequency of each ARG class is also shown.

As reported by other authors, viromes harbored a huge amount of phage-encoded bacterial sequences (López-Bueno et al., 2009; Rosario et al., 2009; Roux et al., 2012; Fancello et al., 2013; Modi et al., 2013). The functional classification analysis carried out on viromes and microbiomes highlights interesting differences in the relative abundance of the different gene classes. Whereas the three microbiomes showed a very similar distribution for each gene functional class, the three viromes showed potential differences. The reads associated with genes coding for functions related to the microbial metabolism apparently decreased from sample A (37%) to sample C (15%), which was enriched with reads associated with genes coding for nucleotide transport and metabolism category (56% compared to 11 and 14% in samples A and B, respectively). The ecological reasons for these differences are not known, but the bacterial genes present in the viromes surely reflect the gene content harbored in the genomes of the bacterial taxa subjected to viral lytic cycles. Concerning the abundance of ARGs- and MRGs-associated reads (abundance of ARGs and MRGs reads on the total amounts of reads) (**Figure 8**), we apparently observed their increase in the three microbiomes from samples A to C and an opposite trend in the three viromes (**Figure 8**).

The increase of ARGs and MRGs-associated reads from samples A to C could be coinciding with the increased industrialization/urbanization level of the geographical area where samples were collected. This association, if confirmed by further studies, is in agreement with a previously formulated hypothesis that considers the exposure of environmental landscapes to pollutants (antibiotics, metals, and disinfectants) a risk for the selection and appearance of multidrug resistant strains (Berendonk et al., 2015). Moreover, a link between heavy metals and the maintenance/acquisition of antibiotic resistances has been documented (Knapp et al., 2011; Chen et al., 2015; Teixeira et al., 2016). This correlation is caused by cross- and co-resistance phenomena. Cross-resistance occurs when the same mechanism reduces the susceptibility to antibiotics and metals simultaneously, whereas co-resistance happens when different resistance mechanisms (ARGs and MRGs) are co-localized in the same genetic locus, and therefore they are co-transferred in case of horizontal gene transfer mechanisms (Baker-Austin et al., 2006). The contamination of the Lambro River with heavy metals is well documented (Camusso et al., 1995; Galli et al., 1998; Barghigiani et al., 2001; Farkas et al., 2007; Viganò et al., 2008), and the toxic effects of these pollutants are also well known (Tchounwou et al., 2012). In biological systems, heavy metals have been reported to affect cellular structures, several physiological activities (Wang and Shi, 2001), and metal ions have been found to cause DNA damage. Therefore, the presence of heavy metals in water environment represents a selective pressure that could force the co-selection of MRGs and ARGs in the microbiome in the absence of antibiotic molecules or in the presence of low concentrations of antibiotic molecules. In this context, it is worth mentioning the increased relative abundance of mobilome-associated reads in the viromes of sample A, thus increasing the probability of transduction events in that environment.

In a previous study carried out on an aquaculture water sample, we observed that microbial ORFs identified in the virome did not reflect the microbiome taxonomy, as determined by *16S rRNA* gene profiling or shotgun metagenomic analysis, suggesting that microbial genes mobilized in the genome of viruses could be considered to be remnants of past recombination events rather than a picture of the current microbial diversity (Colombo et al., 2016). Likewise, in this study we observed the same phenomena (**Figure 3**) in all three samples analyzed, thereby confirming that viromes represent a picture of past microbial populations.

To understand whether the microbial genes associated with the viromes were accessible to members of the aquatic microbiota, we carried out a set of mesocosms focusing our attention on the mobilization of *tet* genes. We observed significant changes in the abundance of *tet* genes in viromes collected from mesocosms exposed to low dose of antibiotic compared to the abundance of these genes in the control mesocosms not exposed to tetracycline. These data are not sufficient to demonstrate gene mobilization from virome to microbiome as a consequence of the selective pressure introduced using a low dose of tetracycline, but they represent the first evidence of the modulation of specific genes within viral populations as a consequence of a specific environmental perturbation. Further studies are necessary in order to understand the mechanisms that drive the gene flow between microorganisms and the complex viral populations.

## Conclusion

We have developed an integrated approach suitable for the exploration of microbiomes and viromes for microbial genes with a focus on ARGs and MRGs starting from fresh water river samples. This approach could represent a useful tool for the characterization of phage-bacterial ecological network in water environments, and for the understanding of environmental constrains which are relevant in the mobilization of ARGs and MRGs.

## Author Contributions

SC and GG have carried out the bioinformatic analysis, SC performed the virome isolation. SC, GD, and EN carried out the mesocosms-based and the plate-count experiments. SA, GD, and EN have analyzed the data. DM and SC have planned the experimental design and they wrote the manuscript.

## Conflict of Interest Statement

The authors declare that the research was conducted in the absence of any commercial or financial relationships that could be construed as a potential conflict of interest.

## Footnotes

1 http://prodigal.ornl.gov/

2 http://www.bioinformatics.babraham.ac.uk/projects/fastqc/

3 http://hannonlab.cshl.edu/fastx_toolkit/

4 http://homer.salk.edu/homer/ngs/homerTools.html

5 ftp://ftp.ncbi.nlm.nih.gov/blast/db/
