# Supplementary material for: Viromes As Genetic Reservoir for the Microbial Communities in Aquatic Environments: A Focus on Antimicrobial-Resistance Genes
Source: Front Microbiol. 2017 Jun 15;8:1095. doi: 10.3389/fmicb.2017.01095 (PMC5471338; doi:10.3389/fmicb.2017.01095)
Supplement: Supplementary file 1 [file Table_1.DOCX]

| **Parameter** | **Viral metagenomic shotgun (sample A)** | **Viral metagenomic shotgun (sample B)** | **Viral metagenomic shotgun (sample C)** | **Microbial metagenomic shotgun (sample A)** | **Microbial metagenomic shotgun (sample B)** | **Microbial metagenomic shotgun (sample C)** |
| --- | --- | --- | --- | --- | --- | --- |
| Technology | MiSeq (Illumina) | MiSeq (Illumina) | MiSeq (Illumina) | MiSeq (Illumina) | MiSeq (Illumina) | MiSeq (Illumina) |
| No, of reads Pre QC | 2132186 | 2108872 | 1884248 | 1980983 | 2504030 | 2239970 |
| No, of reads Post QC | 1923826 | 1667022 | 1663419 | 1945185 | 2344177 | 2028886 |
| Minimum length (nt) | 35 | 35 | 35 | 35 | 35 | 35 |
| Maximum length (nt) | 251 | 251 | 251 | 251 | 251 | 251 |
| Average lenght (nt) | 248 | 249 | 249 | 246 | 247 | 246 |
| Mean G+C | 45 | 51 | 40 | 53 | 50 | 47 |
| No. Of READS annotated with NCBI | 241571 | 104209 | 92897 | 580717 | 426559 | 210576 |
| No. Of READS annotated with NCBI_Viral_DB | 368703 | 140738 | 125177 | \ | \ | \ |
| No, of ORFs predicted | 6822 | 14260 | 30539 | 15420 | 10213 | 10980 |
| Minimum length (aa) | 20 | 20 | 20 | 20 | 20 | 20 |
| Maximum length (aa) | 2866 | 2417 | 2431 | 1400 | 1202 | 2643 |
| Average lenght (aa) | 171.73 | 131.46 | 145.51 | 151.58 | 150.15 | 143.63 |

TABLE S1 Next-generation sequencing metadata, including assembly and annotation statistics.
