# Supplementary material for: Viromes As Genetic Reservoir for the Microbial Communities in Aquatic Environments: A Focus on Antimicrobial-Resistance Genes
Source: Front Microbiol. 2017 Jun 15;8:1095. doi: 10.3389/fmicb.2017.01095 (PMC5471338; doi:10.3389/fmicb.2017.01095)
Supplement: Supplementary file 2 [file Table_2.DOCX]

|  |  |  |
| --- | --- | --- |

TABLE S2 Relative abundance of reads associated to MRGs and biocide resistant genes

|  | Microbial sample | | | Viral sample | | |
| --- | --- | --- | --- | --- | --- | --- |
|  | sample A | sample B | sample C | sample A | sample B. | sample C |
| 2-nitroimidazole [class: imidazole] | 0,48% | 0,70% | 0,69% | 0,02% | 0,43% | 0,61% |
| 6-dichloroindophenol [class: Phenolic Compounds] | 0,10% | 0,07% | 0,06% | 0,03% | - | - |
| Aluminium (Al) | 0,13% | 0,19% | 0,20% | 0,05% | 0,33% | 4,36% |
| Antimony (Sb) | 0,72% | 0,74% | 0,99% | 0,57% | 1,12% | 5,69% |
| Arsenic (As) | 2,83% | 3,15% | 3,23% | 3,28% | 3,62% | 6,90% |
| Benzylkonium Chloride (BAC) [class: Quaternary Ammonium Compounds (QACs)] | 0,02% | 0,02% | 0,02% | - | - | - |
| Bismuth (Bi) | 0,08% | 0,07% | 0,07% | 0,05% | 0,18% | 0,61% |
| Cadmium (Cd) | 4,86% | 4,76% | 4,96% | 3,00% | 5,00% | 2,66% |
| Cetylpyridinium Chloride (CPC) [class: Quaternary Ammonium Compounds (QACs)] | 0,48% | 0,70% | 0,69% | 0,02% | 0,43% | 0,61% |
| Chromium (Cr) | 2,52% | 3,24% | 2,95% | 0,27% | 2,35% | 3,75% |
| Cobalt (Co) | 6,94% | 6,66% | 6,83% | 2,27% | 6,27% | 3,75% |
| Copper (Cu) | 12,80% | 12,32% | 12,75% | 10,18% | 20,46% | 15,62% |
| Crystal Violet [class: Triarylmethane] | 1,53% | 1,11% | 1,14% | 0,33% | 0,43% | 0,24% |
| Cyclohexane [class: Cycloalkane] | 0,22% | 0,15% | 0,14% | 0,47% | 0,07% | 0,12% |
| Dequalinium [class: Quaternary Ammonium Compounds (QACs)] | - | - | - | 0,18% | - | - |
| Diphenyl Ether [class: Phenyl] | 0,05% | 0,04% | 0,04% | 0,05% | - | 0,12% |
| Dodine [class: Acetate] | 0,51% | 0,71% | 0,72% | 0,02% | 0,43% | 0,61% |
| Ethidium Bromide [class: Phenanthridine] | 0,03% | 0,59% | 0,56% | 0,34% | 0,22% | - |
| Gallium (Ga) | 2,09% | 1,97% | 2,17% | 2,44% | 1,09% | 1,33% |
| Glycerol [class: Alcohol] | 0,12% | 0,08% | 0,09% | 0,12% | 0,22% | - |
| Gold (Au) | 1,65% | 1,23% | 1,30% | 0,25% | 0,47% | 0,24% |
| Hydrochloric acid (HCl) [class: Acid] | 0,30% | 0,32% | 0,35% | 0,12% | 0,07% | 0,12% |
| Hydrogen Peroxide (H2O2) [class: Peroxides] | 1,43% | 1,58% | 1,60% | 3,82% | 1,34% | 4,12% |
| Iron (Fe) | 8,13% | 7,73% | 8,01% | 15,56% | 5,90% | 8,23% |
| Lead (Pb) | 1,59% | 1,75% | 1,58% | 0,67% | 1,67% | 0,73% |
| Magnesium (Mg) | 1,18% | 1,13% | 1,25% | 0,77% | 1,01% | 0,97% |
| Manganese (Mn) | 2,44% | 2,00% | 2,15% | 7,17% | 1,23% | 3,39% |
| Menadione [class: Naphthoquinone] | 0,06% | 0,11% | 0,06% | - | - | 0,12% |
| Mercury (Hg) | 1,57% | 1,93% | 1,78% | 4,05% | 1,38% | 2,30% |
| Methyl Viologen [class: Paraquat] | 0,09% | 0,12% | 0,08% | 0,18% | - | 0,12% |
| Methylene Blue [class: Thiazinium] | 1,57% | 1,18% | 1,19% | 0,15% | 0,47% | 0,36% |
| Methylmercury Acetate [class: Organo-mercury] | 0,06% | 0,04% | 0,04% | 0,24% | 0,04% | - |
| Molybdenum (Mo) | 3,11% | 3,13% | 2,90% | 5,09% | 2,32% | 2,18% |
| n-hexane [class: Alkane] | 0,05% | 0,04% | 0,04% | 0,05% | - | 0,12% |
| Nickel (Ni) | 7,37% | 7,11% | 7,07% | 12,77% | 7,68% | 4,72% |
| Pentane [class: Alkane] | 0,05% | 0,04% | 0,04% | 0,05% | - | 0,12% |
| Phenylmercury Acetate [class: Organo-mercury] | 0,69% | 0,77% | 0,71% | 1,74% | 0,22% | 0,97% |
| Plumbagin [class: Naphthoquinone] | 0,01% | 0,01% | 0,01% | 0,00% | 0,04% | - |
| Proflavin [class: Acridine] | 0,03% | 0,04% | 0,05% | 0,18% | 0,07% | - |
| Selenium (Se) | 1,57% | 2,02% | 1,79% | 1,03% | 1,67% | 5,45% |
| Silver (Ag) | 2,86% | 3,31% | 3,12% | 2,87% | 8,51% | 1,09% |
| Sodium acetate [class: Acetate] | 0,13% | 0,13% | 0,12% | 0,65% | 0,14% | 0,12% |
| Sodium azide [class: Azide] | 0,17% | 0,13% | 0,18% | - | 0,07% | 0,12% |
| Sodium Chenodeoxycholate [class: Acid] | 0,58% | 0,58% | 0,54% | 0,15% | 0,22% | - |
| Sodium Cholate [class: Acid] | 0,58% | 0,58% | 0,54% | 0,15% | 0,22% | - |
| Sodium Deoxycholate (SDC) [class: Acid] | 1,38% | 1,29% | 1,20% | 1,08% | 1,16% | 0,36% |
| Sodium Deoxycholate [class: Acid] | 0,58% | 0,58% | 0,54% | 0,15% | 0,22% | - |
| Sodium Dodecyl Sulfate (SDS) [class: Organo-sulfate] | 0,58% | 0,58% | 0,54% | 0,15% | 0,22% | - |
| Sodium Taurocholate [class: Acid] | 0,58% | 0,58% | 0,54% | 0,15% | 0,22% | - |
| Tellurium (Te) | 2,37% | 2,36% | 2,46% | 0,92% | 2,06% | 5,21% |
| Tetraphenylphosphonium (TPP) [class: Quaternary Ammonium Compounds (QACs)] | - | - | - | 0,18% | - | - |
| Tributyltin (TBT) [class: Organo-tin] | 0,01% | 0,01% | 0,01% | 0,99% | - | - |
| Triclosan [class: Phenolic compounds] | 0,54% | 0,59% | 0,60% | 0,04% | 0,29% | 0,36% |
| Tungsten (W) | 5,20% | 5,29% | 4,86% | 6,98% | 3,88% | 2,91% |
| Vanadium (V) | 0,65% | 0,71% | 0,77% | 0,05% | 0,33% | 0,48% |
| Zinc (Zn) | 14,31% | 13,33% | 13,33% | 7,80% | 14,02% | 8,11% |
