# Supplementary material for: Viromes As Genetic Reservoir for the Microbial Communities in Aquatic Environments: A Focus on Antimicrobial-Resistance Genes
Source: Front Microbiol. 2017 Jun 15;8:1095. doi: 10.3389/fmicb.2017.01095 (PMC5471338; doi:10.3389/fmicb.2017.01095)
Supplement: Supplementary file 3 [file Table_3.docx]

| Sample | n. of ribosomal reads on the total of reads in virome |
| --- | --- |
| Spring (present study) | 0,0354% |
| Elbarbera (Fancello *et al.*, 2013) | 0,0211% |
| Reclaimed water (Rosario et al., 2009) | 0,0189% |
| Antartic open soil (Zablocki et al., 2014) | 0,0095% |
| Hamdoun (Fancello *et al.*, 2013) | 0,0076% |
| Indian Ocean GS108 (Williamson et al., 2012) | 0,0069% |
| Antarctic lake summer (López-Bueno *et al*., 2009) | 0,0026% |
| Ponte Lambro (present study) | 0,0009% |
| Antartic hypolithic soil (Zablocki et al., 2014) | 0,0008% |
| Milan (present study) | 0,0006% |

TABLE S3: Level of bacterial contamination in viromes from public databases.
